# How Do Internal and External Control Factors Affect Cyberbullying? Partial Test of Situational Action Theory

**DOI:** 10.3390/bs15070837

**Published:** 2025-06-20

**Authors:** Seong-Sik Lee, Sohee Jung

**Affiliations:** 1 Department of Information Sociology, Soongsil University, Seoul 06978, Republic of Korea; lss824@ssu.ac.kr; 2 Department of Criminology and Criminal Justice, University of South Carolina, Columbia, SC 29208, USA

**Keywords:** cyberbullying, situational action theory, internal and external control, self-control, deterrence

## Abstract

This study attempts to provide a comprehensive explanation for cybercrimes, with emphasis on cyberbullying, by applying situational action theory (SAT). Various hypotheses regarding the motivational and moral dimensions of cyberbullying are presented. Specifically, the interaction effects between motivational and moral factors, such as individual morality and environmental factors of differential association with cyberbullying peers, are examined. Moreover, the roles of self-control and deterrence are investigated as internal and external control factors in situations where conflicts arise between an individual’s morality and the moral rules of their environment. The findings of this study support the assertions of SAT and demonstrate significant interaction effects between cyberbullying victimization and moral factors. Furthermore, consistent with SAT’s discussion on conflicts in the moral dimension, this study reveals that self-control functions as a control factor in situations where individuals possess high morality but are confronted with high levels of differential association with cyberbullying peers; however, the argument that deterrence operates in situations of low differential association with cyberbullying peers and low individual morality is not supported. Despite the partial verification of SAT, this theory is generally endorsed and offers utility in explaining cyberbullying.

## 1. Introduction

Owing to the pervasive adoption of the Internet and smartphones, cyberbullying has become an increasingly common experience among children, adolescents, and college students. Cyberbullying is defined as “willful and repeated^1^ harm inflicted through the medium of electronic text” (Patchin & Hinduja, 2006, p. 152) and encompasses flaming, harassment, denigration, impersonation, outing, trickery, exclusion, and cyberstalking (Willard, 2007). To understand the characteristics of cyberbullying perpetration, prior studies have investigated various predictive factors, e.g., strain, delinquent peer association, and low self-control, derived from major criminological theories (Hinduja & Patchin, 2008; Patchin & Hinduja, 2011; Kowalski et al., 2014; Marcum et al., 2014; Curry & Zavala, 2020; S. S. Lee et al., 2021; G. Lee et al., 2023).

However, these theories alone typically fail to provide sufficient explanations for cyberbullying, thus suggesting the necessity for a comprehensive and new theoretical approach. This study applies situational action theory (SAT) (Wikström, 2004, 2010) as an integrated attempt for explaining crime. SAT, while being a comprehensive theory that attempts to account for the complex relationship of various factors, has not been extensively investigated in cyberbullying. The characteristics and content of SAT suggest its potential applicability to cyberbullying. Specifically, SAT is an integrated theory that addresses the interaction between individual and environmental factors in explaining criminal behavior. It encompasses a wide range of factors including criminal motivation, morality, and control. As suggested by previous studies, cyberbullying involves certain motivations, moral norms, and morality, coupled with weak internal and external controls (Perren & Gutzwiller-Helfenfinger, 2012; Marcum et al., 2014; Li et al., 2016; S. S. Lee et al., 2021). SAT is highly relevant in this context because it comprehensively addresses these factors.

SAT and its discussion of moral dimensions emphasize the interaction between an individual’s morality and the moral rules of their environment, thereby highlighting the process by which individuals perceive and make choices in such contexts. It asserts that the likelihood of criminal behavior is high when individual morality and moral environmental rules are low. However, in situations of incongruence, where an individual possesses high morality but encounters low moral environmental rules, or vice versa, SAT posits that self-control (internal control) and deterrence (external control) are crucial in explaining criminal behavior. Thus, SAT offers complex and novel arguments that extend beyond the aforementioned theories.

This study tests the key propositions of SAT by examining whether internal control (self-control) is more important than external control (deterrence) in preventing cyberbullying perpetration, and the manner by which moral factors interact with these control factors to influence cyberbullying perpetration. In particular, this study investigates the potential effectiveness and limitations of interventions by the police and other criminal-justice authorities in deterring cyberbullying by assessing the role of external control (deterrence). We begin by providing a brief overview of SAT and reviewing previous studies conducted in this area. Subsequently, we present and validate various hypotheses and discuss the suitability of SAT in explaining and preventing cyberbullying.

## 2. Literature Review

### 2.1. SAT and Previous Studies

SAT (Wikström, 2004, 2010) is a prominent integrative theory that explains criminal behavior. This theory strongly emphasizes the interaction between individuals and their environment when elucidating criminal conduct. In contrast to prior discussions that isolated individual factors or solely emphasized environmental factors, this theory seeks to unify individual and environmental elements. It highlights the relationship between individuals and their environments within the context of criminal situations, where such situations are defined as the process by which actors perceive and select alternatives to their actions. In essence, it posits that in criminal situations, both individual factors (e.g., criminal tendencies) and environmental factors (e.g., the criminogenic environment) interact with each other, thus ultimately resulting in an individual’s perception and selection of actions (criminal behavior).

Within this theory, significant importance is placed on the motivational aspect of individuals, thus suggesting that an individual’s temptation and provocation cause him/her to engage in criminal activities. Notably, these personal motives are considered necessary but not sufficient conditions for criminal behavior. This theory asserts that individual motivations must pass through moral filters. The SAT fundamentally views crime as a moral act. Most importantly, it accentuates moral dimensions as being paramount. It posits that an individual’s moral character, in terms of their morality, interacts with moral rules in their environment, thus shaping an individual’s perception of whether their actions are morally acceptable, and influencing their decisions regarding criminal choices. Moreover, this theory contends that when individuals with certain motivations encounter a deficiency in their moral character or moral rules in their environment, they are more likely to opt for criminal activity (Wikström et al., 2012).

This theory extends its focus beyond the moral dimension to emphasize control-related factors, where internal control (self-control) and external control (deterrence) are distinguished as key elements. However, these control factors are proposed to operate primarily when discord exists between an individual’s morality and the moral rules in their environment. In other words, SAT posits that control factors function when an individual’s morality is strong but the surrounding moral environment is weak, or vice versa. For instance, when an individual has a high level of morality but is embedded in a criminogenic environment with weak moral rules, then self-control (internal control) contributes significantly to criminal decision making. Conversely, when the environmental moral rules are strong but an individual’s moral character is weak, then deterrence (external control) becomes crucial in explaining criminal behavior (Wikström, 2010).

Previous studies have predominantly focused on aspects of SAT that are related to the moral dimension, particularly how moral factors interacted with self-control and deterrence as control factors. For instance, as SAT posits that self-control becomes a significant explanatory factor in cases where individuals with high moral characters encounter environments with low moral rules, studies have been performed previously to verify this assertion. Several studies have supported this claim by demonstrating that low self-control is a critical explanatory factor in criminal behavior, particularly in environments marked by contact with delinquent peers (Qusey & Wilcox, 2007; Meldrum et al., 2013; Hirtenlehner et al., 2015). Some studies suggest that self-control serves as a control factor when an individual’s morality is low (Craig, 2019; Hirtenlehner & Kunz, 2016; Schoepfer & Piquero, 2006; Svensson et al., 2010). However, other studies demonstrated the insignificant effect of the interaction between morality and self-control (Antonaccio & Tittle, 2008; Gallupe & Baron, 2014; Song & Lee, 2020).

Furthermore, SAT asserts that deterrence contributes significantly to explaining criminal behavior when environmental moral rules are strong but an individual’s moral character is weak (Wikström, 2010). However, few studies have specifically addressed the effects of punishment or deterrence in situations involving conflicts between individual morality and environmental moral rules. Previous studies showed that the deterrent effect of punishment was particularly evident among individuals with low morality (Hirtenlehner & Hardie, 2016; Svensson, 2015; Wikström et al., 2011; B. R. Wright et al., 2004). However, other studies showed that the interaction between morality and deterrence was not statistically significant (Gallupe & Baron, 2014; Pauwels et al., 2011).

Most existing studies have not examined whether self-control or deterrence serves as a control factor in situations where individual morality conflicts with environmental moral rules, as proposed by SAT. Pauwels (2018) tested whether self-control operated in such conflicting situations. His findings suggest that self-control helps restrain violent behavior not only when both individual morality and environmental moral rules are weak but also when individual morality is weak but environmental moral rules are strong. Consequently, his results do not support SAT. According to Hirtenlehner and Hardie (2016), deterrence (external control) is relevant when individual morality is weak, whereas self-control (internal control) is relevant when environmental moral rules are weak.

### 2.2. Cyberbullying: Previous Studies

#### 2.2.1. Cyberbullying Motivator: Cyberbullying Victimization

Although studies that apply SAT to the topic of cyberbullying are scarce, studies addressing the effects of key factors outlined in the aforementioned theory are prevalent. Among the primary motivational factors in cyberbullying research, previous experiences of cyberbullying victimization have been regarded as a significant motivator and root cause. According to the general strain theory (Agnew, 1992), experiencing negative emotions as a result of strain in daily life contributes to crime. Victimization is one of the factors of strain (Agnew, 2006). When individuals have previously experienced victimization, they can develop negative emotions, and as a coping or resolution mechanism, they may engage in harmful behaviors toward others. Additionally, victims may not only seek relief but also engage in retaliation and vengeance against their aggressors. Cyberbullying victims typically experience anger and react to their negative experiences of victimization (Patchin & Hinduja, 2006). Prior research has asserted that experiencing cyberbullying victimization is a major motivating factor for cyberbullying perpetration (M. F. Wright & Li, 2013; Marcum et al., 2014; M. F. Wright, 2016; S. S. Lee et al., 2021).

#### 2.2.2. Moral Factors: Differential Association with Cyberbullying Peers and Morality

Within the moral dimension of SAT, differential association with bullying peers is considered a significant factor related to moral norms in the environment. Differential association with delinquent peers has long been identified as one of the most influential factors in delinquency and crime research (Akers, 1985; Warr & Stafford, 1991). In cyberbullying research, differential association with peers has been evaluated as a crucial explanatory factor (Marcum et al., 2014; Li et al., 2016; G. Lee et al., 2023). When close friends experience cyberbullying, individuals are more likely to learn and imitate such behavior, or within a peer-group context, perceive bullying as acceptable or even encouraged. This increases the likelihood of engaging in cyberbullying.

Another moral dimension is individual morality, in particular ethical attitudes. In online environments, anonymity, low levels of face-to-face interaction, and weakened normative structures can erode individuals’ ethical standards, thus rendering morality a critical explanatory factor in cyberbullying (Perren & Gutzwiller-Helfenfinger, 2012; Bussey et al., 2015). In response to social pressure and impulsive situations, individuals with strong morality can exert control over their cyberbullying behavior even when confronted with bullying motives (S. S. Lee et al., 2021). Thus, individual morality serves as a key protective factor against cyberbullying and can interact with other motivating factors such as bullying victimization.

#### 2.2.3. Internal and External Control Factors: Self-Control and Deterrence

Self-control, which is a key control factor emphasized in SAT, has long been acknowledged as a major cause of criminal activity in the general theory of crime (Gottfredson & Hirschi, 1990). Individuals with low self-control are evaluated as having a high likelihood of engaging in criminal behavior (Pratt & Cullen, 2000). Additionally, self-control has been shown to buffer the criminogenic effects of strain, such as victimization (Hay & Evans, 2006; Turanovic & Pratt, 2013; Boccio & Beaver, 2021), as well as the influence of delinquent peer associations (Meldrum et al., 2013; Hirtenlehner et al., 2015; Hirtenlehner & Hardie, 2016).

In the context of cyberbullying, high self-control functions as a protective factor that mitigates various motivations and risky situations, whereas low self-control increases the risk of cyberbullying perpetration (Donner et al., 2014; Lianos & McGrath, 2018; Marcum et al., 2014). Additionally, the moderating role of self-control has been identified in cyberbullying research (S. S. Lee et al., 2021; Wang & Ge, 2021).

Deterrence, which is another control factor proposed in SAT, has traditionally been regarded as a major factor in crime prevention. According to deterrence theory (Zimring & Hawkins, 1973; Gibbs, 1975), individuals are deterred from offending when the perceived costs of punishment imposed by official agencies outweigh the benefits of criminal behavior. However, prior deterrence studies have yielded mixed findings, where the effect of punishment perception on crime has been shown to be weak or inconclusive (Nagin, 2013; Pratt et al., 2006). Nevertheless, some studies suggest that the deterrent effects of punishment are conditional and more pronounced among individuals with low morality (Pauwels et al., 2011; Svensson, 2015). Furthermore, the deterrence effect can vary depending on the presence of delinquent peers. Hirtenlehner (2019) discovered that deterrence is more effective when individuals have delinquent close friends, whereas Matthews and Agnew (2008) discovered stronger deterrent effects among individuals with law-abiding close friends.

In investigating cyberbullying, Patchin and Hinduja (2018) examined the perceived deterrent effects of punishment from the police, schools, and parents. Their findings indicated that the deterrent influence of the police was weaker than that of schools and parents. However, Hsieh et al. (2023) showed that, among middle-school youths in Taiwan, the perceived risk of punishment imposed by the police, along with schools and parents, significantly reduced cyberbullying behavior. Similarly, S. S. Lee (2006) suggested that perceived punishment exerts a moderate but significant influence in reducing cyberbullying. However, the explanatory power of deterrence is lower for cyberbullying compared with other types of cybercrime (S. S. Lee, 2018).

## 3. Current Study and Hypotheses

In this study, we aim to validate several hypotheses using SAT as a framework for explaining cyberbullying.

First, SAT posits that criminal motivations influence criminal behavior through a moral filter. Thus, one can argue that cyberbullying motivations are likely to interact with individual morality or the moral-rule environment, which are considered moral-filter factors. This argument resembles the integrated proposition of general strain theory (GST), which suggests that motivation exists and crime is more likely to occur in an environment where moral values are low or the environment is conducive to crime (Agnew, 1992; Piquero & Sealock, 2000). Based on this hypothesis, cyberbullying is more likely to occur in situations where both motivation and low morality or low moral rules coexist.

The first hypothesis aims to verify whether people with high cyberbullying motivations are more likely to commit cyberbullying if the individual morality is low and the environmental moral rules are weak. Hypotheses 1-1 and 1-2 suggest that those who have experienced cyberbullying victimization are more likely to commit cyberbullying when their personal morality is low and their differential association with cyberbullying peers is strong.

**Hypothesis** **1-1.** *Individuals with experience of cyberbullying victimization are more likely to commit cyberbullying when their morality is low; the interaction effect between victimization and morality is negatively significant.*

**Hypothesis** **1-2.** *Individuals with experience of cyberbullying victimization are more likely to commit cyberbullying when differential association with cyberbullying peers is high; the interaction effect between victimization and differential association with cyberbullying peers is positively significant.*

Second, SAT discusses control factors in addition to moral dimensions. It suggests that self-control and deterrence factors operate when conflict exists between individual morality and the moral-rule environment. However, previous studies have focused solely on the interaction between individual morality and self-control (Antonaccio & Tittle, 2008; Svensson et al., 2010), the interaction effect between the criminogenic environment and self-control (Hirtenlehner et al., 2015), or the interaction effect between low morality and deterrence (Wikström et al., 2011; Svensson, 2015). Thus, a more detailed examination of the operation of control factors in situations involving individual–environmental dimension conflicts is necessitated.

According to SAT, the control mechanism operates when the moral filters of individual morality and environmental moral rules do not match and conflict with each other. In other words, when the morality of the individual is high, but the environmental moral rules are weak, self-control will serve as a control factor; when the environmental moral rules are strong, but the morality of the individual is low, the deterrent of official punishment will serve as a control factor.

The second hypothesis is formed to test whether people with high self-control are less likely to commit cyberbullying if their morality is high but their differential association with cyberbullying peers is strong. Additionally, it seeks to test whether people with high deterrence (perceived punishment) are less likely to commit cyberbullying if their morality is low but their differential association with cyberbullying peers is weak.

**Hypothesis** **2-1.** *Individuals with high individual morality but weak environmental moral rules (strong differential association with cyberbullying peers) with respect to cyberbullying are less likely to commit cyberbullying when self-control is high; the interaction effect between differential association with cyberbullying peers and self-control is negatively significant, particularly when morality is high.*

**Hypothesis** **2-2.** *Individuals who have strong environmental moral rules (weak differential association with cyberbullying peers) but low personal moral standards with respect to cyberbullying are less likely to commit cyberbullying when the deterrence (perceived punishment) is high; the interaction effect between morality and punishment inhibition is positively significant, particularly when the differential association with cyberbullying peers is low.*

## 4. Data and Methods

### 4.1. Sample and Procedure

This study focused on cyberbullying through smartphone use among college students. We used data obtained from a 2017 survey of college students and targeted individuals attending seven universities in Seoul. A total of 266 participants were surveyed, with 30–40 students from each school. We applied a quota-sampling method that considered academic level, major, and gender. For example, if 40 participants were assigned to a particular university, then the target distribution would include 20 men and 20 women. Within each gender group, the participants were further balanced by major (10 from engineering/natural sciences and 10 from humanities/social sciences) and academic level to include two to three students per year from freshman to senior. This final stage of recruitment was performed using a non-probability quota-sampling method, in which participants were selected to match predefined quotas based on their gender, major, and academic level. Data from 250 smartphone users were analyzed. Regarding the participants’ sociodemographic characteristics, the sample included 135 males (54.0%) and 115 females (46.0%). Participants ranged in age from 18 to 27 years, with a mean age of 21.96 years (SD = 2.07).

### 4.2. Measurements

For the dependent variable of this study, cyberbullying was defined based on Willard’s (2007) discussion and a cyberviolence survey conducted by the Korea Internet and Security Agency. Specifically, we investigated the participants’ experiences of perpetrating cyberbullying over the past year based on the following six categories: (1) insults, (2) defamation/false information dissemination, (3) stalking, (4) sexual harassment, (5) disclosure of personal information, and (6) exclusion. The respondents were asked to indicate the frequency of involvement in each category as “none,” “once,” “2–3 times,” “4–9 times,” or “10 times or more.” The responses were scored from 0 to 4, and the scores were summed (alpha = 0.889; McDonald’s ω = 0.914).

To measure the motivation for cyberbullying as an independent variable, we assessed the experiences of cyberbullying victimization over the past year based on the aforementioned six categories. The participants rated the victimization frequency in each category as “none,” “once,” “2–3 times,” “4–9 times,” or “10 times or more.” The responses were scored from 0 to 4, and the scores were summed (alpha = 0.811; McDonald’s ω = 0.834).

Morality was assessed by posing six questions to the participants regarding whether they believed it was wrong to engage in each of the six cyberbullying behaviors. The participants responded on a 5-point scale, i.e., “not at all (=1),” “no,” “normal,” “agree,” and “strongly agree (=5),” and their responses were summed (alpha = 0.957; McDonald’s ω = 0.960).

Differential association with cyberbullying peers was measured by asking the participants whether they had friends who experienced the six cyberbullying behaviors. The participants reported the number of friends in each category as “none,” “1 friend,” “2–3 friends,” “4–9 friends,” or “10 friends or more.” The responses were scored from 0 to 4, and the scores were summed (alpha = 0.867; McDonald’s ω = 0.902).

Self-control was assessed based on six characteristics, namely impulsivity, risk-taking, simple task preference, activity, competitiveness, and irritability, based on prior research (Grasmick et al., 1993). Each characteristic was measured relative to one item, such as “I typically act impulsively,” and the participants responded on a 5-point scale, i.e., “not at all (=1),” “no,” “normal,” “agree,” and “strongly agree (=5).” Because higher scores on all six items reflected lower self-control, we reverse coded all items so that higher scores would indicate higher self-control. The reverse-coded items were then summed to create the final self-control scale (alpha = 0.632; McDonald’s ω = 0.637).

Deterrence of punishment was measured by posing six questions to the participants regarding whether they believed they would be punished for each of the six cyberbullying behaviors. The participants responded on a 5-point scale, i.e., “not at all (=1),” “no,” “normal,” “agree,” and “strongly agree (=5),” and their responses were summed (alpha = 0.912; McDonald’s ω = 0.915).

As for the sociodemographic control variables, gender was coded as female (=0) and male (=1), birth year was inquired and recoded into age, and the participants’ subjective economic status ranged from “very low (=1)” to “very high (=5).”

### 4.3. Analytic Strategies

First, we investigated whether people with cyberbullying victimization as a cyberbullying motivation are more likely to commit cyberbullying when their personal morality is low and their differential association with cyberbullying peers is strong. For Hypotheses 1-1 and 1-2, we tested the interaction effects (*cyberbullying victimization* × *morality* and *cyberbullying victimization* × *differential association with cyberbullying peers*) on cyberbullying perpetration. Second, we investigated whether people with high self-control are less likely to commit cyberbullying if their morality is high but their differential association with cyberbullying peers is strong. Additionally, we examined whether people with high deterrence are less likely to commit cyberbullying if their morality is low but their differential association with cyberbullying peers is weak. To test the hypotheses, after we categorized morality into high and low levels centered on the average, we tested whether the interaction effect (*differential association with cyberbullying peers × self-control*) can be confirmed when morality is high. Similarly, after we categorized the differential association with cyberbullying peers into high and low levels centered on the average, we tested whether the interaction effect (*morality × deterrence*) can be confirmed when the differential association with cyberbullying peers is low.

We conducted an ordinary least-squares (OLS) regression analysis using SPSS 24.0 while controlling for sociodemographic variables. The interaction terms were mean-centered to mitigate multicollinearity. Key assumptions of OLS regression were assessed. First, the residual-versus-fitted values plot revealed a curved, non-random pattern, suggesting potential violations of the linearity assumption. Second, multicollinearity diagnostics indicated no serious concern, with all variance inflation factors (VIFs) below 2.0 (mean VIF = 1.41). Third, tests for normality (Shapiro–Wilk and skewness–kurtosis) rejected the null hypothesis (*p* < 0.001), but the residual histogram appeared approximately symmetric and centered around zero, with minor deviations driven by a few outliers—supporting the assumption of approximate conditional normality. Finally, influence diagnostics (Cook’s distance and leverage plots) suggested that most observations were within acceptable limits, though a few high-leverage cases may require cautious interpretation. Taken together, these diagnostics indicate that while the OLS regression model is generally estimable, several assumptions are statistically violated. Specifically, the presence of non-linearity suggests that standard errors and *p*-values derived from conventional OLS may be biased.

Nonetheless, we conducted OLS regression analyses to estimate interaction effects, following prior SAT research that prioritized the interpretability and comparability of conditional relationships (Hardie & Rose, 2025). Hardie and Rose (2025), systematically reviewed empirical studies testing the situational model of SAT, and offered several methodological recommendations to advance SAT research. One of their key arguments is that most existing empirical studies are based on non-situational-level data (e.g., survey data) rather than observational data from real-life crime settings. In such contexts, researchers typically rely on statistical interaction terms to approximate the theoretical idea of “moral contexts.” Importantly, the authors highlight a methodological pattern: studies using parametric methods such as OLS regression are more likely to detect significant interaction effects compared with those using non-parametric methods (e.g., negative binomial or Tobit models). They argue that this discrepancy is not merely a statistical artifact but reflects the challenges of modeling interaction effects, especially under skewed data conditions. Rather than discarding parametric methods, they recommend that researchers clearly report their methodological decisions and, where feasible, apply multiple modeling approaches to cross-validate findings. In line with their recommendation, we employed OLS regression while acknowledging and reporting its limitations, especially with respect to data skewness and the interpretative nuances of statistical interaction effects.

## 5. Results

The subjective economic status of their families was measured on a 5-point scale ranging from 1 to 5, with a mean of 3.15 (SD = 1.23), indicating that most participants perceived themselves to be of moderate to slightly above average economic standing. Regarding the main variables of this study, cyberbullying victimization, which was considered a motivational factor, recorded a mean score of 3.28 within the range of 0–24. Individual morality, which represents the moral dimension, exhibited a notably high average score of 28.00 within the range of 6–30. Differential association with cyberbullying peers, which reflects the moral-rule environment, recorded a mean score of 1.78 within the range of 0–24. In terms of the control factors, self-control recorded a mean score of 18.45 within the range of 6–30, whereas deterrence (perceived punishment) recorded an average of 24.24 within the same range. Finally, the dependent variable, i.e., cyberbullying perpetration in smartphone usage, recorded a considerably low mean score of 1.880 within the range of 0–24 (See Table 1).

The skewness and kurtosis values suggest that several variables, particularly morality, differential association with cyberbullying peers, cyberbullying victimization, and cyberbullying perpetration, substantially deviate from normality, indicating non-normal distributions. In contrast, variables such as self-control (skewness = 0.36, kurtosis = 0.60) and deterrence (skewness = –0.83, kurtosis = 1.43) exhibited values closer to normal thresholds. These distributional patterns were further supported by the Shapiro–Wilk normality test, which indicated that all variables significantly deviated from a normal distribution (*p* < 0.05). In particular, morality, differential association with cyberbullying peers, deterrence, and cyberbullying perpetration demonstrated substantial non-normality, consistent with their high skewness and kurtosis values. Although the Shapiro–Wilk statistics for self-control (W = 0.904) and cyberbullying victimization (W = 0.983) were statistically significant, their test statistics were closer to 1.0, suggesting approximate normality.

Table 2 shows the zero-order correlations among the study variables. Cyberbullying perpetration was significantly and negatively correlated with morality (r = −0.262) and self-control (r = −0.169). In contrast, cyberbullying perpetration showed significant positive correlations with differential association with cyberbullying peers (r = 0.688) and cyberbullying victimization (r = 0.746). The correlation between deterrence and cyberbullying perpetration was not statistically significant (r = −0.083).

First, in accordance with the assertions of SAT, as presented in the first hypothesis, the influence of cyberbullying victimization and the manner by which such motivations result in cyberbullying when the moral dimensions include low morality and low moral-rule environments was examined, as illustrated in Table 3. The results revealed that cyberbullying victimization exerted a significant positive effect on cyberbullying perpetration (β = 0.400, *p* < 0.001), while individual morality demonstrated a significant negative effect (β = −0.078, *p* < 0.05). However, the independent impact of differential association with cyberbullying peers was not statistically significant.

Importantly, the results of the interaction effects support Hypotheses 1-1 and 1-2. The interaction effect between cyberbullying victimization and morality was negatively significant (β = −0.157, *p* < 0.001), indicating that the effect of victimization on perpetration decreases among individuals with higher moral standards. Conversely, the interaction between cyberbullying victimization and differential association with cyberbullying peers was positively significant (β = 0.350, *p* < 0.001), suggesting that victimization leads to more cyberbullying when individuals are embedded in peer environments that tolerate or support such behavior. Furthermore, the standardized coefficient for the interaction between victimization and cyberbullying peers was larger than that of victimization and morality, indicating that peer influence may amplify the effect of victimization more strongly than personal morality mitigates it.

The results were analyzed to understand the functions of self-control and deterrence in cases where a conflict exists between individual morality and the moral-rule environment, as presented in Table 4a,b. For these analyses, we categorized participants into high (upper-level) and low (lower-level) morality groups in Table 4a, and into high and low differential association with cyberbullying peers in Table 4b. Although the morality scores were clustered at the upper end (M = 28 on a 6–30 scale; skewness = −3.40), we used a split at the mean to distinguish between relatively high (≥28) and low (≤27) morality groups. For differential peer association, the distribution was also highly skewed (M = 1.78 on a 0–24 scale; skewness = 2.764). Accordingly, participants were categorized into high (≥1.78) and low (≤1.77) peer association groups. These cut-off points were selected to capture relative variation within the scales and to enable meaningful contrasts in interaction analyses. Moreover, splitting at the mean allowed us to retain sufficient sample sizes in both groups, avoiding the limitations of extreme or percentile-based splits that could have resulted in imbalanced or underpowered comparisons.

As shown in Table 4a, we examined the interaction effects between differential association with cyberbullying peers and self-control, specifically testing whether individuals with high self-control are less likely to commit cyberbullying when their personal morality is high but their peer environment is criminogenic. Consistent with the discourse on SAT, the results revealed that among participants with high morality, the interaction between differential association with cyberbullying peers and self-control was significantly negative (β = −0.201, *p* < 0.05). This finding indicates that the criminogenic effect of associating with cyberbullying peers is mitigated by self-control only when individuals possess high levels of moral commitment. In other words, self-control functions as an internal control mechanism in morally committed individuals when exposed to criminogenic environments. This supports Hypothesis 2-1.

As shown in Table 4b, we examined the interaction effects between individual morality and deterrence, specifically testing whether individuals with higher perceived deterrence are less likely to commit cyberbullying when their individual morality is low but their peer environment is non-criminogenic. However, the interactions were not statistically significant in either peer group (β = 0.151 in the high-peer group; β = −0.062 in the low-peer group). Contrary to the expectation of Hypothesis 2-2, deterrence did not demonstrate a moderating effect on cyberbullying perpetration under conditions of low morality and weak association with cyberbullying peers. That is, even in less criminogenic peer environments, external controls such as perceived punishment did not appear to buffer the influence of low morality on cyberbullying behavior. This finding raises questions about the conditional relevance of deterrence mechanisms in cyberbullying contexts.

## 6. Discussion and Conclusions

This study applied SAT as an integrated framework to explain cyberbullying. Various hypotheses were proposed based on SAT, and the analysis of data acquired from university students in Seoul, South Korea, generally supported the SAT’s propositions. First, the findings showed that the influence of motivational factors on cyberbullying as well as their interaction with morality and differential association with cyberbullying peers were significant. Individuals with strong cyberbullying motivations were more likely to engage in cyberbullying when their individual morality was low or when their association with cyberbullying peers was strong.

Furthermore, the results provided partial support regarding SAT’s proposition that self-control and deterrence operate as control mechanisms when conflict exists between individual morality and environmental moral rules. Specifically, self-control was shown to mitigate cyberbullying in contexts characterized by high morality and high differential association with cyberbullying peers, thus aligning with SAT’s expectations. However, the hypothesis that deterrence operates effectively when individual morality is low and environmental moral rules are strong was not supported.

These findings suggest that, in the context of cyberbullying prevention and intervention, internal controls such as self-control may be more important than external controls such as harsher punishment. This aligns with previous studies that highlight the limited effectiveness of deterrence; perceptions of punishment showed only a weak deterrent effect, even among individuals with low morality (Nagin, 2013; Pratt et al., 2006). Similarly, our results indicated that deterrence exerted marginal influence on cyberbullying behavior, thus suggesting that punitive approaches may not be effective on their own. However, the deterrent effect may be better captured through a macro-level approach focused on broader societal moral conditions instead of by considering micro-level environmental factors such as differential association with lawbreakers. Accordingly, deterrence may be effective primarily when individuals with low morality are placed within a morally sound and cohesive society. From this perspective, the weak deterrent effects observed in this study may stem from focusing on micro-level environmental moral factors instead of macro-level societal moral climates. As this study restricted its operationalization of environmental morality to peer-level associations, it cannot fully assess how larger societal moral contexts might influence the operation of deterrence; therefore, definitive conclusions regarding SAT’s full applicability could not be inferred. Future studies should expand the measurement frameworks to include broader environmental variables, such as perceptions of societal moral consensus or community-level normative climates.

In summary, whereas the discussion of control factors in the moral dimensions of SAT was only partially validated, the theory received empirical support in general. Moreover, the findings underscore the fact that both individual and environmental moral dimensions, which operate both independently and interactively with motivational factors, are critical in explaining cyberbullying. This highlights the importance of fostering strong moral values and attitudes to prevent cyberbullying, as emphasized in previous studies (Perren & Gutzwiller-Helfenfinger, 2012; Bussey et al., 2015; Marcum et al., 2014; Li et al., 2016). Thus, SAT remains a highly useful theoretical framework for understanding and addressing cyberbullying, and future studies should further explore its application.

### 6.1. Limitations and Future Research

However, this study has several limitations. First, limitations related to the sample and generalizability should be noted. The sample comprised university students in South Korea, which may have limited the generalizability of the findings. Future studies should examine broader populations, including Western and younger individuals, such as middle- and high-school students. Second, some measurement issues may have affected the reliability and validity of key constructs. The internal consistency of the self-control scale was relatively low, as we used a reduced version of Grasmick et al.’s (1993) scale with only six items instead of the original 24 items. Moreover, morality scores were highly skewed, which may reflect social desirability bias and potentially compromise construct validity.

Third, the measurement of deterrence focused solely on the perceived certainty of punishment across different types of cyberbullying, limiting its explanatory power. The recent literature suggests that deterrence is better conceptualized as the product of both perceived certainty and severity of punishment (Sattler et al., 2022). Future studies should incorporate both dimensions. In addition, it may be important to consider informal sanctions, such as reputational costs or social exclusion, which can serve as meaningful deterrents, especially in settings where discrepancies exist between personal and contextual moral norms (Antonaccio et al., 2017). Although Antonaccio and colleagues focused on offline contexts, the role of informal sanctions may be especially relevant in cyberbullying, where anonymity and weak legal enforcement reduce the salience of formal punishment, while social consequences may exert more immediate behavioral influence. Fourth, this study operationalized environmental moral rules at the micro level (i.e., differential association with cyberbullying peers), which constrains our ability to assess SAT’s full claims regarding the role of broader moral contexts in settings. Future work should incorporate macro-level indicators, such as a perceived societal moral consensus or a community-level moral climate, to more comprehensively evaluate SAT’s claims regarding the contextual operation of deterrence and self-control.

Lastly, the analysis relied on self-reported survey data collected in non-situational formats, which limits the extent to which the full situational dynamics proposed by SAT can be tested. In particular, while we examined statistical interactions between morality and environmental moral contexts, the study design did not capture the real-time perception-choice processes emphasized in SAT’s situational model. As noted by Hardie and Rose (2025), most empirical applications of SAT are constrained by the level of data and design; individual-level survey data can only approximate the types of moral–contextual interactions envisioned by the theory. Thus, while the findings offer meaningful insight into how control factors operate under moral conflict conditions, the absence of significant interaction effects should not be interpreted as definitive evidence against the theory. Future research should use more situation-sensitive designs to reflect the conditional mechanisms central to SAT.

### 6.2. Conclusions and Implications

This study advances cyberbullying research by empirically testing key propositions of situational action theory (SAT). Specifically, it analyzes how self-control and deterrence function under moral conflict conditions in cyberbullying contexts. Results showed that self-control significantly buffered the criminogenic effects of peer influence among individuals with strong moral commitment, supporting SAT’s emphasis on internal moral regulation. In contrast, perceived deterrence failed to moderate cyberbullying behavior even among individuals with low morality and non-criminogenic peer environments. The null findings may reflect both the theoretical limitations of deterrence in digital contexts, where sanctions are often seen as delayed, inconsistent, or unlikely. By evaluating the applicability of SAT to digital behavior, this study clarifies which control strategies are most effective in preventing cyberbullying and informs future interventions beyond traditional punitive approaches.

Based on the current findings, future interventions should prioritize strengthening self-control and moral reasoning among the youth. Public campaigns and school-based initiatives should not only aim to raise awareness about cyberbullying (Amarah et al., 2020) but also cultivate digital empathy, ethical reflection, and positive peer influence. Considering the significant role of differential association with cyberbullying peers, school-based interventions should target peer group norms by fostering positive peer influence and collective accountability via online interactions (Álvarez-Turrado et al., 2024). In addition to moral education, schools should implement socio-emotional learning programs that strengthen self-control, emotional regulation, and impulse management (Mohamed Othman et al., 2025). Moreover, policies that provide comprehensive support systems for victims, including clinical interventions, legal advice, and social-support networks, are essential. Research, prevention, and intervention efforts should be expanded and systematically evaluated to determine strategies that most effectively reduce cyberbullying and facilitate evidence-based policy development.

## Figures and Tables

**Table 1:** Descriptive Statistics and Kolmogorov–Smirnov Test Results for Study Variables.

Variable	Mean	S.D.	S.E	Skewness	Kurtosis	Range	S–W Statistic	*p*-Value
*Economic Level*	3.145	1.233	0.077	−0.897	2.172	1–5	0.519	<0.001
*Morality*	28.000	4.223	0.265	–3.402	13.167	6–30	0.598	<0.001
*Differential Association with Cyberbullying Peers*	1.779	3.347	0.208	2.764	8.238	0–24	0.776	<0.001
*Cyberbullying Victimization*	3.281	4.121	0.258	1.742	2.713	0–24	0.983	<0.001
*Self-control*	18.445	3.772	0.236	0.364	0.600	6–30	0.904	0.005
*Deterrence*	24.237	4.561	0.284	−0.826	1.428	6–30	0.551	<0.001
*Cyberbullying Perpetration*	1.880	3.751	0.234	3.017	9.625	0–24	0.519	<0.001

Note. S.D. = Standard Deviation; S.E. = Standard Error of the Mean; S–W = Shapiro–Wilk.

**Table 2:** Zero-order Correlations of the Variables.

Variable	1	2	3	4	5	6
*Morality*	−					
*Differential Association with Cyberbullying Peers*	−0.210 *	−				
*Cyberbullying Victimization*	−0.195 *	0.682 *	−			
*Self-Control*	0.092	−0.257 *	−0.239 *	−		
*Deterrence*	0.283 *	−0.029	−0.108	−0.016	−	
*Cyberbullying Perpetration*	−0.262 *	0.688 *	0.746 *	−0.169 *	−0.083	−

Note: * *p* < 0.05 (two-tailed test).

**Table 3:** OLS analysis results of interaction effects between cyberbullying victimization and moral factors on cyberbullying perpetration.

	Cyberbullying Perpetration	
	b	β
*Male*	0.752	0.099
*Age*	−0.097	−0.054
*Economic Level*	0.067	0.022
*Cyberbullying Victimization*	0.368 ***	0.400
*Morality*	−0.072 *	−0.078
*Cyberbullying Peers*	0.061	0.052
*Victimization × Morality*	−0.029 ***	−0.157
*Victimization × Cyberbullying Peers*	0.040 ***	0.350
R square	0.697	
F score	52.725 ***	

Note. * *p* < 0.05, ** *p* < 0.01, *** *p* < 0.001 (two-tailed test).

**Table 4:** (**a**) OLS analysis results of influence of self-control in moral conflict pertaining to cyberbullying perpetration; (**b**) OLS analysis results of influence of deterrence in moral conflict pertaining to cyberbullying perpetration.

(**a**)				
	**Cyberbullying Perpetration**			
	**Morality (High)**		**Morality (Low)**	
	**b**	**β**	**b**	**β**
*Male*	0.308	0.067	0.621	0.058
*Age*	−0.004	−0.059	−0.532 *	−0.211
*Economic Level*	−0.040	−0.021	0.097	0.023
*Cyberbullying Peers*	0.424 ***	0.424	0.839 ***	0.656
*Self-Control*	−0.069	−0.115	0.215	0.144
*Cyberbullying Peers × Self-Control*	−0.040 *	−0.201	−0.027	−0.112
R square	0.326		0.556	
F score	12.893 ***		13.958 ***	
(**b**)				
	**Cyberbullying Perpetration**			
	**Cyberbullying Peers (High)**		**Cyberbullying Peers (Low)**	
	**b**	**β**	**b**	**β**
*Male*	–2.069	0.187	0.494	0.105
*Age*	−0.565 *	−0.229	−0.132	−0.118
*Economic Level*	−0.229	−0.059	−0.058	−0.030
*Morality*	−0.327 *	−0.274	−0.103	−0.171
*Deterrence*	−0.050	−0.042	−0.021	−0.043
*Morality × Deterrence*	0.041	0.151	−0.004	−0.062
R square	0.256		0.052	
F score	4.311 ***		1.405	

Note. * *p* < 0.05, ** *p* < 0.01, *** *p* < 0.001 (two-tailed test).

## Data Availability

The data presented in this study are available on request from the corresponding author due to ethical and privacy restrictions.

## Note

1 Nonetheless, whether cyberbullying requires repeated actions is debated, as a single act can affect countless individuals and harm the victim (Baldry et al., 2016).

## Abbreviations

The following abbreviations are used in this manuscript:

GST	General strain theory
SAT	Situational action theory
OLS	Ordinary least squares
